# Modified Intraoral Repositioning Appliance in Complete Bilateral Cleft Lip and Palate

**DOI:** 10.5005/jp-journals-10005-1096

**Published:** 2010-04-15

**Authors:** Pradeep Raghav, NK Ahuja, Subhash Gahlawat

**Affiliations:** 1 Professor, Department of Orthodontics and Dentofacial Orthopedics, Subharti Dental College, Meerut, Uttar Pradesh, India; 2 Professor and Head, Department of Orthodontics and Dentofacial Orthopedics, Subharti Dental College, Meerut, Uttar Pradesh, India; 3 Ex-Postgraduate Student, Department of Orthodontics and Dentofacial Orthopedics, Subharti Dental College, Meerut, Uttar Pradesh, India

**Keywords:** Bilateral cleft lip and palate, Neonatal maxillary orthopedics, Latham’s appliance.

## Abstract

**Objective:**

The purpose of the modified repositioning appliance was to overcome the shortcoming of existing design for repositioning protruded premaxilla in a child with bilateral cleft lip and palate.

**Methods:**

The basic principles of design were similar to Latham’s appliance but the surgical pinning of premaxillary segment was avoided and instead acrylic splint was prepared.

**Conclusions:**

This technique avoids any invasive procedure, is useful to reposition protruded premaxillary segment in bilateral cleft lip and palate cases specifically in child who reports late with deciduous dentition.

## INTRODUCTION

Presurgical orthopedics is routinely required in the management of complete bilateral cleft lip and palate (BCLP) cases, which have markedly protruded premaxillary segment. The initial step in the management of BCLP is to reposition the protruded premaxilla prior to surgical correction. Repositioning protruded premaxilla serves dual advantages; first it prevents excessive tension at suture line following surgical correction of lips and secondly provides psychological benefit to child because of early esthetic improvement.

Any procedure undertaken at neonatal age to remold or reposition the skeletal or soft tissue segments so as to simplify the surgical procedures in a cleft lip and palate case are commonly referred to as ‘neonatal maxillary orthopedics or presurgical orthopedics’. The concept of presurgical neonatal maxillary orthopedics was first introduced by McNeil (1950),^1^ where he utilized an intraoral prosthesis with a head bonnet and extaoral strap for repositioning of protruded premaxillary segment. Since then, arrays of appliances/methods^2-6^ have been introduced to reposition the premaxilla in BCLP cases. Georgiade and Latham (1975)^3^ developed an intraoral premaxillary repositioning appliance, which was later modified by Millard and Latham^7^ and came to be known as intraoral elastic chain premaxillary repositioning appliance (ECPRA) or more frequently Latham’s appliance. This appliance consists of acrylic pads over the maxillary segments connected posteriorly by an expansion mechanism. The premaxil-lary segment is retracted with elastic bands attached to a pin inserted in the premaxillary bone, just anterior to the premaxillovomeral suture. In BCLP patients where commonly posterior alveolar segments are collapsed, Latham’s appliance had an added advantage of achieving posterior expansion along with repositioning of protruded premaxillary segment. Latham’s appliance, inserted on an average at 2-month-of-age, relocates the segments over 3 to 4 weeks.^8^ Removal of the appliance is immediately followed by functional surgery.

The case presented in this article is of a child aged 4 years and 2 months with complete bilateral cleft lip and palate. Neonatal maxillary orthopedics treatment is customarily initiated within first 6 months after birth, but many times we may come across situations where a child reports at an older age with deciduous dentition, therefore compounding the existing problem. Since appliance which uses oral pinning and traction could have caused interference with growth or damage to developing permanent tooth buds, therefore in this case it was decided not to use any pinning of premaxillary segment. Hence, a modified noninvasive repositioning appliance was fabricated for repositioning of protruded premaxillary segment, although the basic principles of design were similar to Latham’s appliance.

## CASE REPORT

**Fig. 1:**
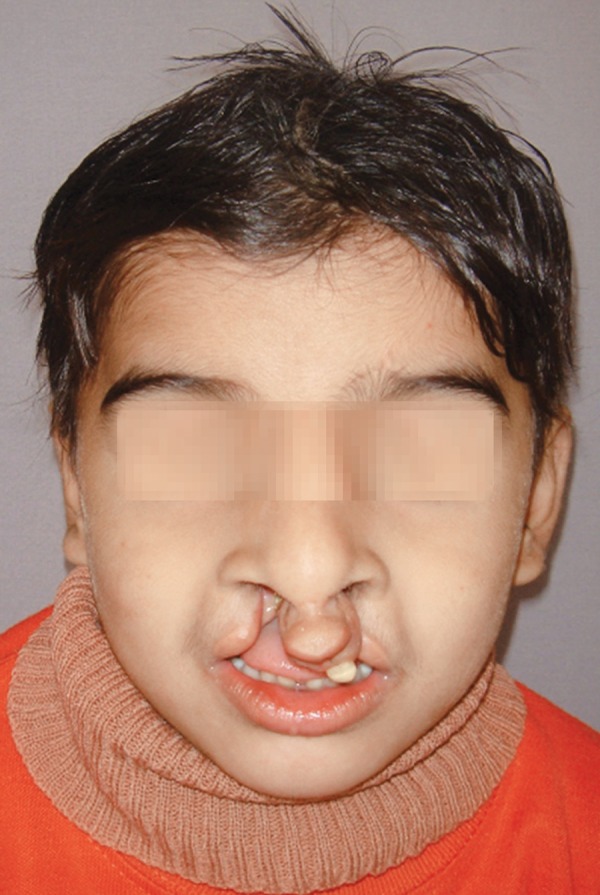
Pretreatment frontal

**Fig. 2:**
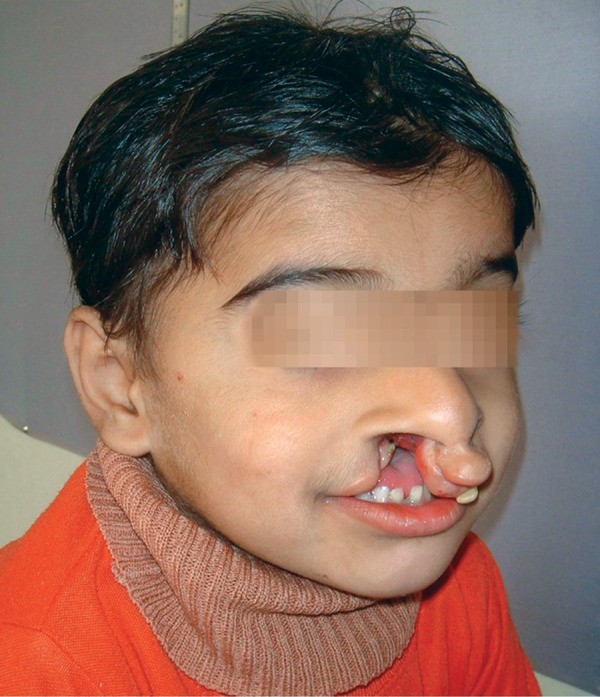
Pretreatment oblique

**Fig. 3:**
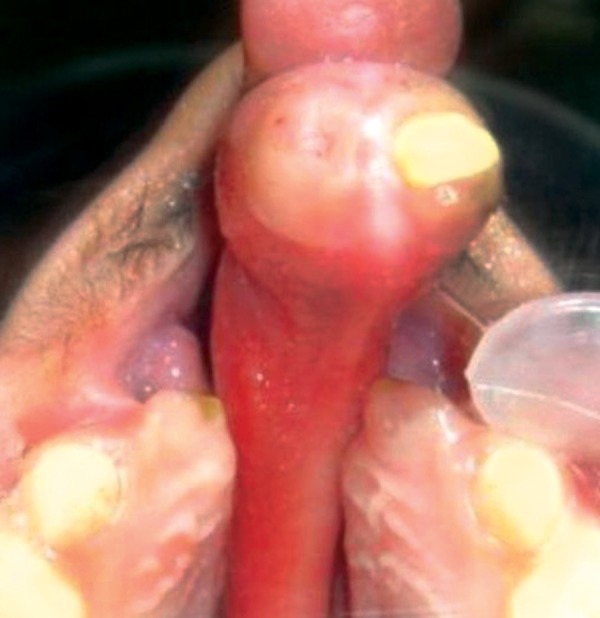
Pretreatment occlusal

The patient Juber aged 4 years and 2 months reported with complete bilateral cleft lip and palate with markedly protruded premaxillary segment shifted to the left side (Figs 1 and 2). Intraoral examination revealed maxillary deciduous canines and molars erupted on both maxillary quadrants, maxillary deciduous lateral incisors were absent, and the premaxillary segment had only erupted left deciduous central incisor (Fig. 3). The maxillary arch width was normal along with markedly protruded premaxillary segment. There was sufficient space to retract the premaxillary segment, therefore, it was decided to use a modified premaxillary repositioning appliance.

## MEASUREMENTS

Measurements were preformed by utilizing following reference points (based on reference points used by Heidbuchel et al).^9^

**Table:** 

**R** Points at which the lateral sulcus crosses the crest of the alveolar ridge	
**R”** Most lateral points of the premaxillae contour on right side	
**L** Points at which the lateral sulcus crosses the crest of the alveolar ridge	
**L”** Most lateral points of the premaxillae contour on left side	

Right and left cleft widths were measured.

Right cleft width (RCW): the distance between R and R”

Left cleft width (LCW): the distance between L and L”.

Following were the recorded measurements on pretreat-ment study models (Fig 9):

**Table:** 

Premaxillary segment width (R” to L”)―21 mm			
Right cleft width (RCW)		20.37 mm	
Left cleft width (LCW)		17.17 mm	

## METHODOLOGY

Dental impressions of the maxillary and mandibular arch were taken using customized impression tray and rubber base impression (polysiloxane) material.

- Impressions were poured in white dental stone (orthocal)
- An acrylic maxillary occlusion split was fabricated on right and left segments, and hooks with 0.9 mm stainless steel wire were placed in the deciduous second molar region on both side, these hooks were directed distally for attachment of right and left elastic bands. A transpalatal arch was incorporated to stabilize the maxillary segments (Fig 4A).
- On the premaxillary segment, model acrylic cap was fabricated with bilateral stainless steel hooks in the most lateral aspect which were directed mesially (Fig 4B).
- A cut was made in this splint for the erupted left deciduous incisor.
- The tissue’s side of the premaxillary splint was relined by perma soft denture reliner for cushioning of sensitive soft tissue.
- The maxillary occlusion splint was cemented on both maxillary segments with GIC cement.
- Orthodontic elastic bands were secured from hooks on maxillary splint to the premaxillary splint applying a force of 200 gm on each side (Fig 5).

**Fig. 4A:**
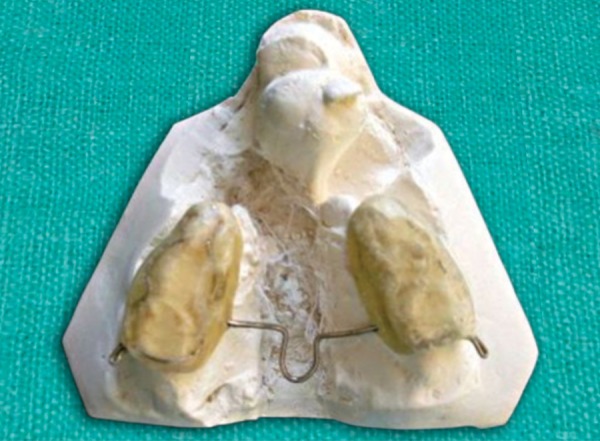
Occlusal splint

**Fig. 4B:**
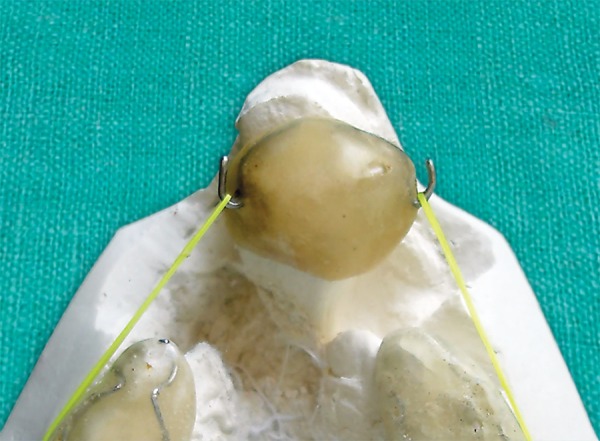
Premaxillary splint

**Fig. 5:**
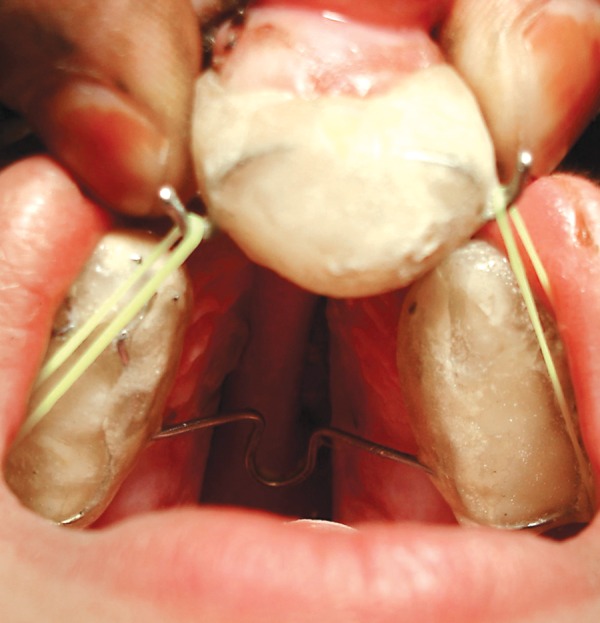
Intraoral view of modified repositioning appliance

**Fig. 6:**
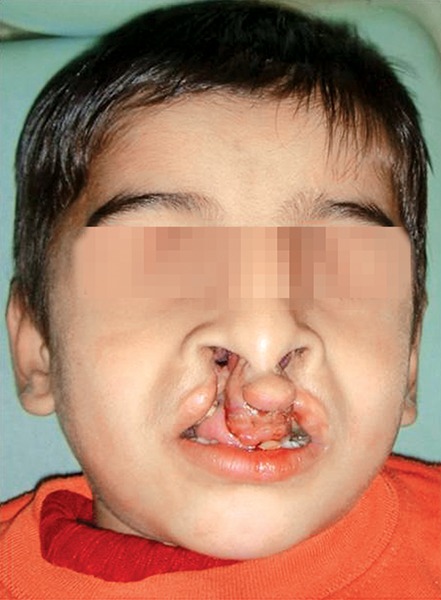
Post-treatment frontal

**Fig. 7:**
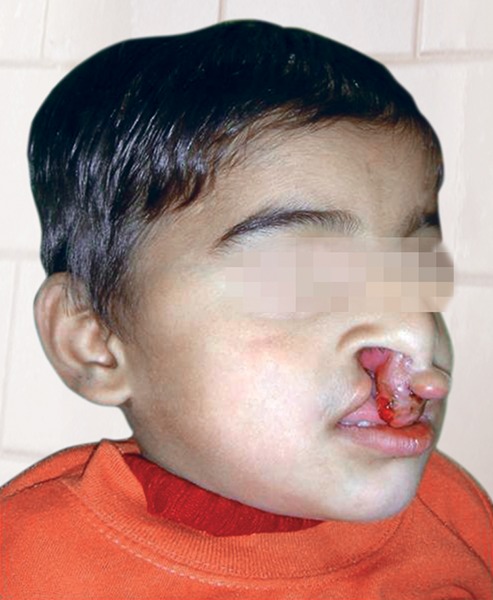
Post-treatment oblique

**Fig. 8:**
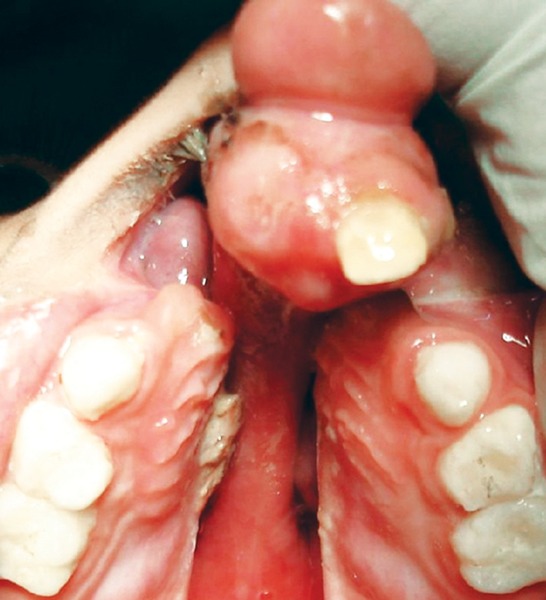
Post-treatment occlusal

The patient was checked weekly and elastic bands were adjusted to reposition and align the premaxillary segment. Within 8 weeks sufficient amount of distal repositioning of premaxillary segment was achieved (Figs 6 to 8).

**Table:** 

		*Pre-* *treatment*		*Post-* *treatment*		*Net reduction* * in cleft width*	
Right cleft width (RCW)		20.37 mm		15.29 mm		5.08 mm	
Left cleft width (LCW)		17.17 mm		12.91 mm		4.28 mm	

After treatment right cleft width was 15.29 mm and left cleft width was 12.91 mm.

Figure 10 shows a distal positioning of 5.08 mm on right side and 4.28 mm on left side.

## DISCUSSION

Since appliance which uses oral pinning and traction can cause interference with growth and may damage developing tooth buds, therefore, in this case it was decided not to use any pinning of premaxillary segment. As the maxillary width was normal, there was sufficient space to retract the premaxillary segment, therefore self expansion of lateral palate was not included in the treatment plan. The premaxillary cap splint was prevented from dislodgement by applying a blob of composite on the labial surface of incisor after placing the splint. Appliance was worn successfully by the patient, following were the problems encountered:

1. Frequent breakage of elastic due to masticatory forces for this parent was trained to change the elastics on daily basis.
2. Patient was recalled every week, and premaxillary segment was removed, cleaned and replaced, at the end of treatment minor bruises were seen.

Controversy exists with regard to treatment and the dental occlusion,^10^ Bitter (1992),^4^ Millard and Latham^8^ believed that these repositioning appliance for alignment of alveolus segment are beneficial not only for lip and nose reconstruction but also for the occlusion as well. Other authors, Bertcovitz (1996),^11^ Henkel and Grundlach (1998)^12^ considered that this results in more malocclusion then when there is no orthopedic treatment.

**Fig. 9:**
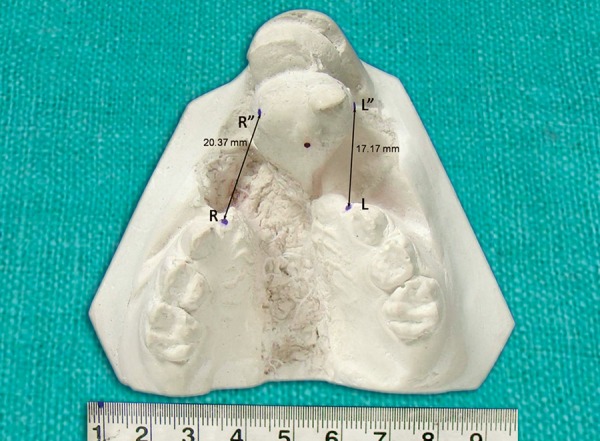
Pretreatment models showing right and left cleft widths

**Fig. 10:**
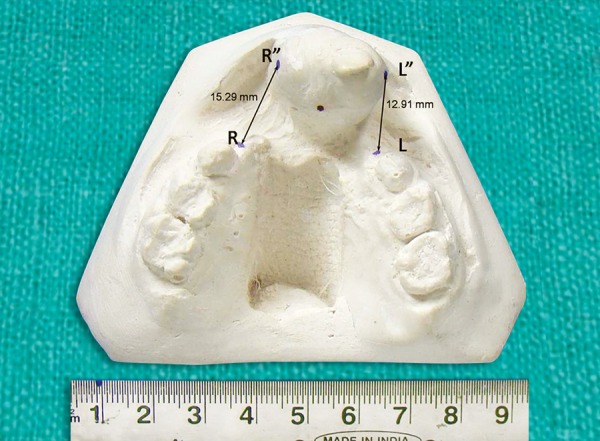
Post-treatment models showing reduction in right and left cleft widths

**Table:** Comparison between Latham’s appliance and modified appliance

*Latham’s appliance*			*Modified appliance*		
1.	Not feasible above		1.	Feasible	
	6 months of age				
2.	Surgical procedures for		2.	No surgical procedures	
	oral pinning may damage			required, therefore	
	developing tooth bud			comparatively safe	
3.	Simultaneous expansion		3.	Not in this appliance but it	
				can be achieved by adding	
				additional wire component	

All considered, the facilitation of lip and nose reconstruction in the difficult case makes presurgical orthopedics with improved technique worthwhile, not only because of reduced tension at the suture line and less need for soft tissue undermining but also because it does eliminate the necessity for additional lip adhesion surgery.
